# Hospital Nursing in War-Time: Disadvantages of Depleted Staffs

**Published:** 1916-06-24

**Authors:** 


### THE EDITOR'S LETTER-BOX.

### Hospital Nursing in War-Time—Disadvantages of Depleted Staffs.

*To the Editor of* The Hospital.

Sir,—Now when so much interest and attention are centred in hospitals, it is unfortunate that complaints should frequently be made of the cavalier treatment which is often meted out to visitors to a hospital by the matron's office staff and the ward sisters. When one seldom hears anything but praise for the nursing of the patients it seems a pity that there should be any lack of civility towards visitors. One way to remedy any fault in this direction would be to employ in the matron's office one or more of the well-educated girls and young women who are longing at the present time for something to do. The chief recommendation and qualification of these ladies should be pleasant manners and common sense; whilst their first duty should be when a stranger enters the office to go forward, to receive her with an appearance of welcome, and, with a sympathetic manner, endeavour to get from her what it is she wants. The visitor's business could then be concisely reported to the matron or matron's assistant, whose answer would be translated into suave language by the "reception clerk." The visitor would then go away pleased with her reception, and determined to do all she can to help this particular hospital, which by this simple means would make many friends for itself.—Yours faithfully,
"Experienced Matron."

[We should like to hear the views of the matrons on this matter. Do they consider there is any ground for the complaints referred to?—Ed. The Hospital.]

